# P75^NTR^ activation limits CD21^lo^ B cell subsets expansion in response to autoimmune-inducing challenges

**DOI:** 10.1016/j.isci.2025.113055

**Published:** 2025-07-03

**Authors:** Cong Luo, An-Hui Zha, Ru-Yi Luo, Zhao-Lan Hu, Wei-Yun Shen, Ru-Ping Dai

**Affiliations:** 1 Department of Anesthesiology, The Second Xiangya Hospital, Central South University, Changsha, China; 2 Anesthesiology Research Institute of Central South University, Changsha, China

**Keywords:** Immunology, Molecular biology, Cell biology

## Abstract

Studies, including our own, suggest that p75^NTR^ plays a pivotal role in immune regulation. Here, we aimed to uncover the role of p75^NTR^ signaling in regulating CD21^lo^ B cell subsets, which are known to facilitate autoimmune activity, and to identify possible regulatory transcripts involved. Through *in vitro* assays, *in vivo* models, and RNA-seq analysis, we found that p75^NTR^ expression and CD21^lo^ B cell expansion were increased in B cells following TLR7/9 stimulation *in vitro* and in pristane-challenged mice *in vivo*. Interestingly, p75^NTR^ deficiency led to a further expansion of CD21^lo^ B cells and enhanced their pro-inflammatory characteristics. RNA-seq data revealed notable transcript alterations associated with CD21^lo^ B cells, including increased *Tbx21* expression. A potential role for p75^NTR^ downstream signaling via phosphorylated p65 (p-p65) was also proposed. Our study provides insights into the role of p75^NTR^ in restraining the development of CD21^lo^ subsets and modulating autoimmune activity in response to autoimmune challenges.

## Highlights

• P75^NTR^ expression increased in B cells following TLR7/9 stimulation
• CD21^lo^ B cells expand with increased activity after TLR7/9 stimulation
• p75^NTR^ loss enhances TLR7/9-induced expansion of CD21^lo^ B cells
• p75^NTR^ may restrain CD21^lo^ B cells via downstream p-p65 signaling

## Graphical abstract

**Figure:**
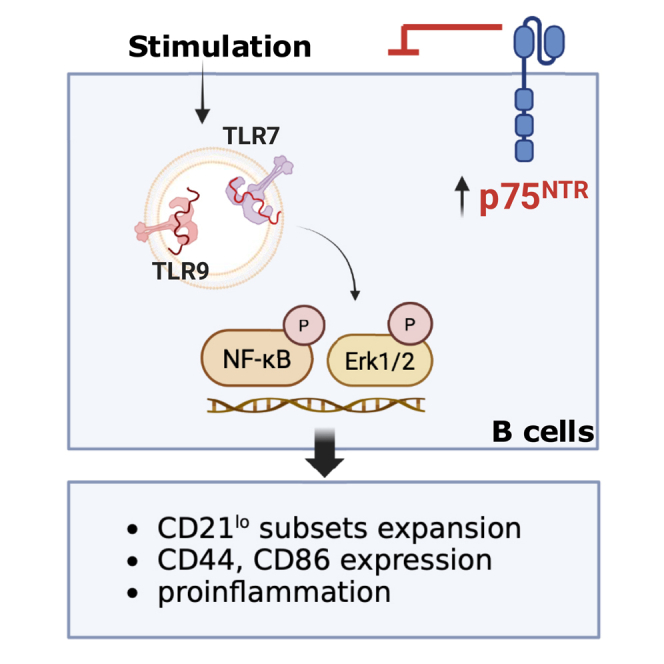

## Introduction

The p75 neurotrophin receptor (p75^NTR^), also known as the nerve growth factor receptor (NGFR), is a pan-neurotrophic receptor that belongs to the tumor necrosis factor (TNF) superfamily.^1^^,^^2^^,^^3^ Within the central nervous system (CNS), p75^NTR^ acts as a high-affinity receptor for pro-neurotrophins, influencing key processes such as neuronal survival, cell death, and synaptic plasticity, depending on its interaction with precursor or mature ligands, like brain-derived neurotrophic factor (BDNF) and its precursor.^4^^,^^5^^,^^6^ Notably, p75^NTR^ is also expressed beyond the nervous system, including in immune cells such as microglia,^7^^,^^8^ macrophages,^9^ neutrophils,^10^ and plasmacytoid dendritic cells,^11^ where it plays a role in regulating inflammation and modulating immune cell function.

Our previous studies have demonstrated the role of p75^NTR^ in modulating the functions of various immune cell subsets. In patients with the autoimmune disease multiple sclerosis, increased expression of p75^NTR^ in lymphocytes facilitated the pathological effects of its high-affinity ligand, the brain-derived neurotrophic factor precursor (proBDNF), contributing to disease progression.^12^ Additionally, p75^NTR^ expression was elevated in activated human peripheral monocytes/macrophages, promoting inflammatory immune responses.^13^^,^^14^ Recently, we identified an upregulation of p75^NTR^ in antibody-secreting B cells (ASCs) in systemic lupus erythematosus. Deficiency of p75^NTR^ in B cells was found to limit ASCs proliferation and differentiation.^15^ Furthermore, a study by Hernandez-Barranco et al. demonstrated that p75^NTR^ acts as an immune tolerance checkpoint in germinal centers (GC); systemic p75^NTR^ knockout led to abnormal GC formation and enhanced follicular dendritic cell and overall B cell activation.^16^ These findings suggest that p75^NTR^ plays a critical role in regulating immune functions and development.

Recent studies have highlighted the specific role of dim CD21 expression on B cells during inflammation.^17^^,^^18^ CD21, also known as complement receptor 2 (CR2), is a cell surface protein primarily expressed on B cells and is crucial for their activation and regulation.^19^^,^^20^^,^^21^ In humans, CD21^lo^ B cells expand under inflammatory conditions and represent an innate-like B cell population, characterized by decreased expression of homeostatic chemokine receptors and increased expression of inflammatory chemokine receptors.^22^ In mice, CD21^lo^ B cells, referred to as age-associated B cells, accumulate with age.^23^ This population of CD21^lo^ B cells expands in several autoimmune diseases,^24^^,^^25^^,^^26^^,^^27^ with toll-like receptors (TLRs) likely being key drivers of this subset.^28^ Specifically, TLR7 and TLR9 are important inducers of CD21^lo^ B cell expansion.^29^^,^^30^ It is well established that TLRs, particularly TLR7 and TLR9, play pivotal roles in driving the development of autoimmunity.^31^^,^^32^^,^^33^ However, whether p75^NTR^ regulates CD21 expression in B cells under TLR stimulation remains unknown.

In this study, we demonstrated that p75^NTR^ expression increases in B cells in response to TLR7/9 stimulation *in vitro* and following pristane injection—a known activator of TLRs in B cells—in vivo. Targeted depletion of p75^NTR^ in B cells led to a further increase in the proportion of CD21^lo^ B cells, accompanied by transcriptomic changes, including upregulation of *Tbx21*, and heightened activation of downstream p75^NTR^ signaling pathways of phosphorylated p65 (p-p65). These findings suggest that p75^NTR^ upregulation in B cells serves a restraining role in the expansion of CD21^lo^ B cell subtypes under autoimmune conditions.

## Results

### P75^NTR^ expression increased in splenic B cells upon stimulation of TLRs *in vitro*

**Figure 1:**
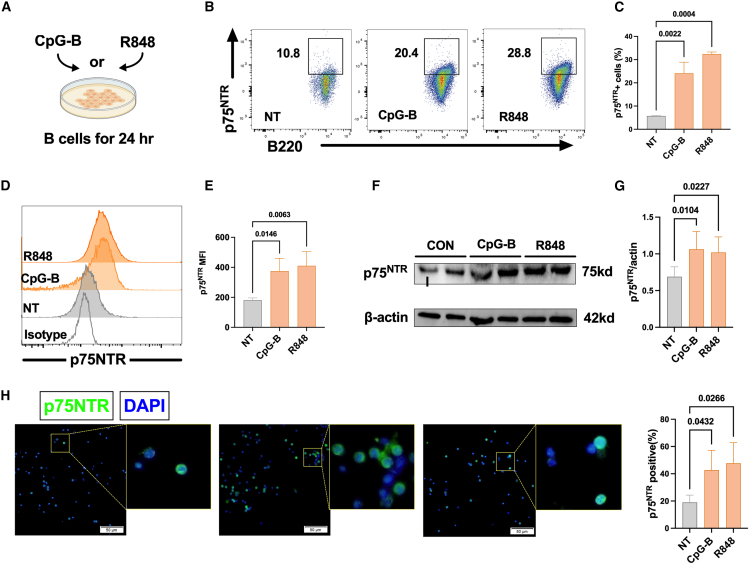
P75^NTR^ expression exhibited an increase in splenic B cells upon stimulation of TLR7/9 *in vitro* (A) B220^+^ splenic B cells from C57BL/6 mice were sorted with magnetic beads and then cultured with CpG-B (0.25 μmol) or R848 (200 ng/mL) for 24 h. (B and C) Representative flow cytometry images and statistical analysis of the proportion of p75^NTR+^ cells in splenic B cells by treatment with CpG-B or R848. (D and E) Representative flow cytometry images and statistical analysis show the changes of p75^NTR^ MFI in splenic B cells after CpG-B or R848 treatment. (F and G) Representative western blot images and statistical analysis of the gray value showing p75^NTR^ expression in splenic B lymphocytes after CpG-B or R848 treatment. (H and I) Immunofluorescence staining and statistical analysis were performed in splenic B cells after CpG-B or R848 treatment. Scale bar, 50 μm. Data are presented as mean ± SEM of three independent experiments, n = 3–6 in each group. One-way analysis of variance (ANOVA) was used for statistical analysis.

To explore the expression of p75^NTR^ in B cells after TLRs activation, we stimulated splenic purified B cells from C57BL/6 mice with TLR9 agonist CpG-B, and TLR7 agonist R848 for 24 h (Figure 1A). Compared with resting B cells, the percentage of p75^NTR+^ cells and the p75^NTR^ mean fluorescence intensity (MFI) was upregulated in B cells both activated by CpG-B and R848 (Figures 1B–1E). Correspondingly, western blot revealed increased protein levels of p75^NTR^ (Figures 1F and 1G) and immunofluorescence indicated broad p75^NTR^ expression in splenic B cells after treatment of CpG-B or R848 (Figures 1H and 1I). Therefore, these results proved an elevation of p75^NTR^ expression in TLR7 or TLR9-activated B cells, which may be involved in regulating B cell immune functions.

### p75^NTR^ deficiency resulted in the expansion of CD21^lo^ B cells and promoted related immune characteristics

**Figure 2:**
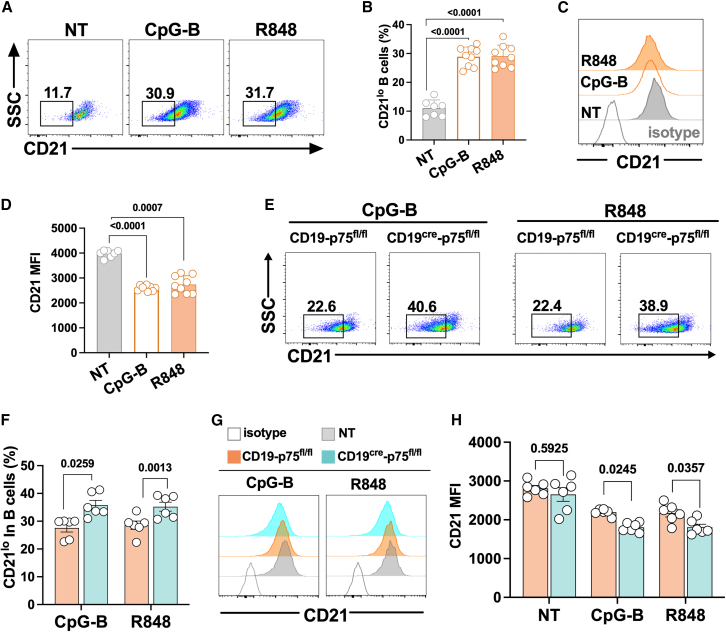
Deficiency of p75^NTR^ induces the expansion of CD21^lo^ subsets and decreases CD21 MFI in B cells (A–D) B220^+^ splenic B cells from C57BL/6 mice were sorted with magnetic beads and then cultured with CpG-B (0.25 μmol) or R848 (200 ng/mL) for 24 h. Representative flow cytometry and quantification showing CD21^lo^ B cell percentages and CD21 MFI in B cells upon CpG-B or R848 stimulation. (E–H) B220^+^ splenic B cells from CD19^cre^-p75^fl/fl^ mice or their CD19-p75^fl/fl^ control were sorted with magnetic beads and then cultured with CpG-B (0.25 μmol) or R848 (200 ng/mL) for 24 h. Representative flow cytometry and statistical analysis showing the changes of CD21^lo^ B cell percentages and the CD21 MFI in B cells. Horizontal bars represent the mean ± SEM of at least three independent experiments. Statistical analyses were performed using one-way ANOVA for Figures 2B and 2D, and unpaired two-tailed Student’s *t* tests for Figures 2F and 2H.

We next want to clarify the role of p75^NTR^ in CD21 expression in B cells upon TLR7/9 stimulation. For that purpose, we used B-cell-specific p75^NTR^ knockout mice as previously described (Figures S1A and S1B). Flow cytometry and western blot confirmed the significant down-regulation of p75^NTR^ protein level in B220^+^ B cells in CD19^cre^-p75^fl/fl^ mice relative to control (Figures S1C–S1E). As shown, the percentage of CD21^lo^ B cells in cultured splenic B cells was increased both upon CpG-B and R848 stimulation (Figures 2A and 2B), in parallel with the CD21 MFI in B220^+^ cells reduced (Figures 2C and 2D). Notably, B cells lacking p75^NTR^ showed a further elevation of CD21^lo^ B cells, which happens similarly upon CpG-B or R848 stimulation (Figures 2E, 2F, and S2). CD21 expression was reduced following CpG-B or R848 treatment, and this reduction was further enhanced in splenic B cells lacking p75^NTR^ under TLR7/9 stimulation (Figures 2G and 2H).

**Figure 3:**
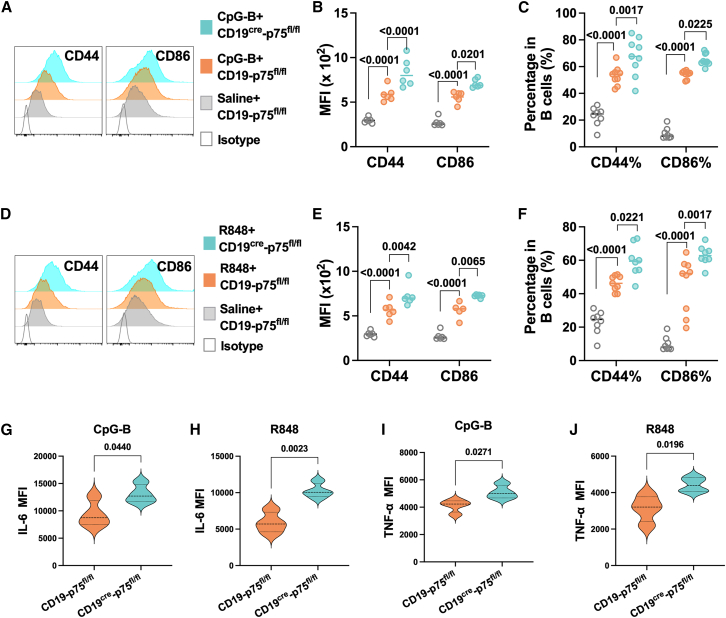
p75^NTR^ deficiency in B cells showing proinflammatory profiles resembling CD21^lo^ B cell subsets. B220^+^ splenic B cells from CD19^cre^-p75^fl/fl^ mice or their CD19-p75^fl/fl^ control were sorted with magnetic beads and then cultured with CpG-B (0.25 μmol) or R848 (200 ng/mL) for 24 h (A–C) Representative flow cytometry histograms and statistical analysis showing CD44 and CD86 MFI (A and B), CD44, and CD86 positive cells (C) in splenic B cells after CpG-B treatment. (D–F) Representative flow cytometry histograms and statistical analysis showing CD44 and CD86 MFI (D and E), CD44 and CD86 positive cells (F) in splenic B cells after R848 treatment. (G–J) LEGENDplex multianalyte flow assays showing IL-6 and TNF-α levels in supernatant from B cells upon CpG-B or R848 treatment. Horizontal bars represent the mean ± SEM of at least three independent experiments. Statistical analyses were performed using unpaired two-tailed Student’s *t* tests.

Increased evidence related to the specific subtype of CD21^lo^ B cells demonstrates its elevated proinflammatory profiles like CD86 and CD44 levels and production of IL-6 and TNF-α, which may exacerbate many inflammatory and autoimmune conditions.^34^ Correspondingly, compared to control, CpG-B or R848 promoted the expression of CD44 and CD86 in splenic B cells from CD19-p75^fl/fl^ mice (Figures 3A, 3B, 3D, and 3E). p75^NTR^-deficient B cells revealed further elevation of CD44 and CD86 after CpG-B or R848 treatment (Figures 3A, 3B, 3D, and 3E). The percentage of CD44^+^ and CD86^+^ cells in splenic B cells also increased after CpG-B and R848 stimulation when deficient of p75^NTR^ (Figures 3C and 3F). P75^NTR^ deficiency in B cells increased expression of CD44 and CD86 in CD21^lo^ B cell subsets (Figure S3). In addition, the multi-analyte flow assay revealed that the MFI of IL-6 in splenic B cells from CD19^cre^-p75^fl/fl^ mice increased after CpG-B or R848 treatment compared to control (Figures 3G and 3H). In parallel, the TNF-α level increased in splenic B cells from CD19^cre^-p75^fl/fl^ mice when stimulated with CpG-B or R848 relative to control (Figures 3I and 3J). Therefore, our results suggest a potential role of p75^NTR^ upregulation in B cells under TLRs stimulation in controlling the differentiation of CD21^lo^ B cell subsets and related proinflammatory responses.

### Deficiency in p75^NTR^ leads to increased CD21^lo^-related transcriptomics and proinflammatory profiles *in vitro* under TLR7/9 stimulation

To further investigate the role of p75^NTR^ in TLR7/9-induced transcriptional regulation in B cells, we conducted RNA-seq to compare the transcriptomic differences in B cells lacking p75^NTR^ upon TLR7 or TLR9 stimulation.

**Figure 4:**
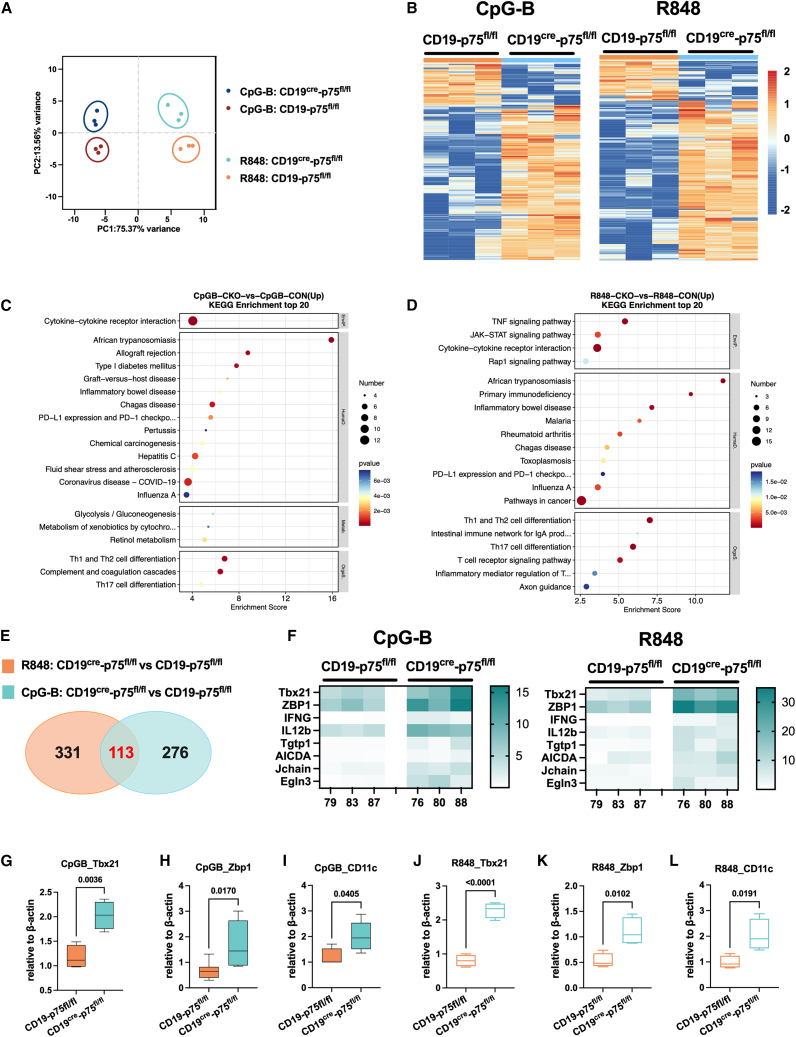
RNA-seq analysis in splenic B cells in mice deficient of p75^NTR^ with CpG-B and R848 stimulation (A) Principal components analysis (PCA) distinguished between CpG-B-stimulated and R848-stimulated differences in splenic B cells and differences in lacking p75^NTR^ in B cells. (B) Heatmap depicting differentially expressed transcripts between CD19^cre^-p75^fl/fl^ mice and the control group with CpG-B and R848 treatment. Red indicates increased relative expression, and blue indicates decreased relative expression. (C and D) Bubble diagram of KEGG enrichment top 20 between CD19^cre^-p75^fl/fl^ mice and control group with CpG-B and R848 treatment. (E) Venn diagram was generated to show an overlap of mapped DEGs from R848 treatment (set orange) and CpG-B (set blue) treatment between CD19^cre^-p75^fl/fl^ mice and the control group. Sets orange and blue share an intersecting set of 113 genes. (F) Heatmap showing the expression patterns of the DEGs between CD19^cre^-p75^fl/fl^ mice and the control group with CpG-B and R848 treatment. (G–L) Quantitative PCR validation of RNA-seq-identified genes (*Tbx21*, *Zbp1*, *Cd11c*) in splenic B cells from CD19^cre^-p75^fl/fl^ vs. control mice after CpG-B or R848 treatment. Horizontal bars represent the mean ± SEM of at least three independent experiments. Statistical analyses were performed using unpaired two-tailed Student’s *t* tests.

Principal component analysis (PCA) showed that principal component (PC) 1 distinguished the effects of CpG-B versus R848 stimulation in splenic B cells, while PC2 captured differences related to p75^NTR^ deficiency in B cells (Figure 4A). The first two components accounted for the majority of transcriptomic variance, but it still clearly shows distinct expression patterns in p75^NTR^-deficient B cells. In CpG-B-stimulated B cells from CD19^cre^-p75^fl/fl^ mice, we identified 389 differentially expressed genes (DEGs), including 170 upregulated and 45 downregulated genes. In R848-stimulated cells, there were 444 DEGs, with 205 upregulated and 51 downregulated (Figures 4B and S4A). KEGG analysis showed that DEGs in CpG-B-activated B cells were enriched in pathways such as cytokine-cytokine receptor interaction, Th1 and Th2 cell differentiation, and complement and coagulation cascades (Figure 4C). Under R848 stimulation, DEGs were associated with TNF and JAK-STAT signaling pathways, Th1 and Th2 cell differentiation, and the intestinal immune network for IgA production (Figure 4D). GO enrichment revealed that DEGs clustered in immune system processes, defense responses to viruses, bacterial responses, and cellular responses to IFN-β upon CpG-B or R848 stimulation in p75^NTR^-deficient B cells (Figures S4B and S4C).

Despite differences between the two ligands, 113 DEGs were shared across both datasets (Figure 4E), suggesting common transcriptional regulations by p75^NTR^ following TLR7 and TLR9 stimulation. Notably, *Tbx21*, a key driver in the development of CD21^lo^ B cells, showed elevated expression in p75^NTR^-deficient B cells (Figure 4F). CD21^lo^ B cells are tissue-homing, innate-like B cells with potential for differentiation into antibody-secreting cells. Among the co-DEGs were genes associated with B cell receptor activity (*Aicda*), antibody production (*Jchain*), and proinflammatory responses (*Zbp1*, *Ifng*, *Il21b*, *Tgtp1*) (Figure 4F). These transcriptional changes indicate that p75^NTR^ deficiency enhances B cell activity and proinflammatory responses under TLR challenges. The increased expression of *Tbx21*, *Zbp1*, and *Cd11c*—typically elevated in CD21^lo^ B cells—were validated in p75^NTR^-deficient B cells upon TLR7/9 stimulation by qPCR (Figures 4G–4L). Therefore, our findings highlight the role of p75^NTR^ in modulating CD21^lo^ B cell expansion in response to immune challenges from DNA or RNA strands.

### Upregulation of p75^NTR^ in B cells restrained the expansion of CD21^lo^ B cell subsets in mice upon pristine treatment

**Figure 5:**
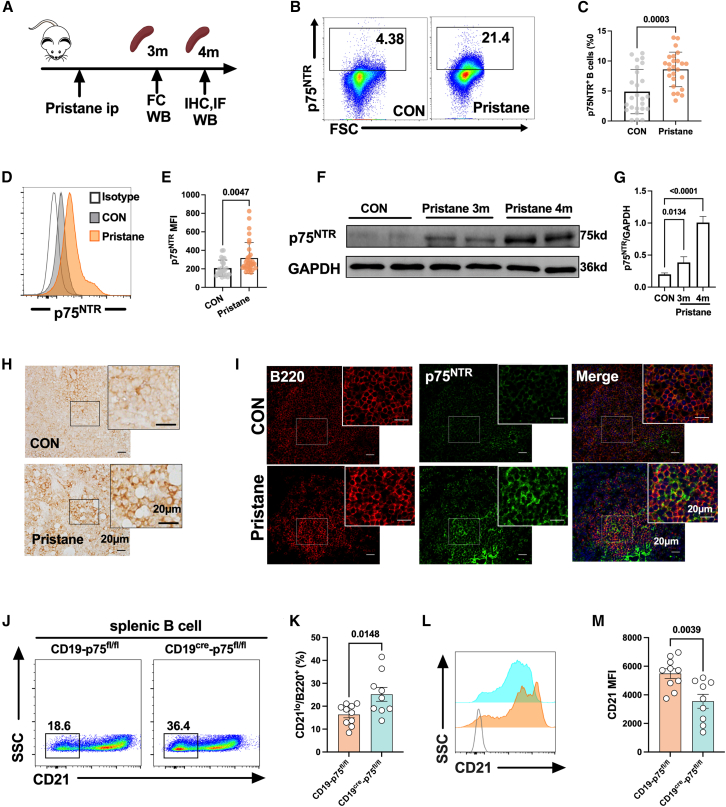
Upregulation of p75^NTR^ in B cells regulates CD21^lo^ B cells in pristane-treated mice (A) Spleens were collected from mice at 3 months or 4 months after pristane injection. (B and C) Flow cytometry plots (left) and statistical analysis (right) show the percentage of p75^NTR+^ B cells in mice after pristane immunization. (D and E) Flow cytometry plots (left) and statistical analysis (right) show p75^NTR^ MFI in B220^+^ B cells in mice after pristane immunization. (F and G) Representative western blot images (left) and statistical analysis of the gray value (right) showing p75^NTR^ expression in splenic lymphocytes from pristane-immunized mice and their corresponding controls. (H–I) Immunohistochemistry (left) and immunofluorescence (right) staining were performed in splenic sections from pristane-injected mice and their controls. Scale bar = 20 μm. (J–M) Representative flow cytometry diagrams and statistical analysis of CD21^lo^ B cells and CD21 MFI in B cells in CD19^cre^-p75^fl/fl^ mice and the control group after pristane immunization. Horizontal bars represent the mean ± SEM of at least three independent experiments. Data were analyzed by one-way ANOVA (Figures 5G–5K and 5M) or unpaired t-tests (Figures 5C–5E).

To confirm our *in vitro* results, we immunized mice with pristane, which induces a robust autoimmune-like response and activates TLR signaling in B cells. In pristane-treated mice, both the proportion of p75^NTR+^ cells and the MFI of p75^NTR^ in splenic B cells were significantly higher compared to control mice (Figures 5A–5E). Western blot analysis also showed markedly increased p75^NTR^ protein levels in the splenic lymphocytes of pristane-treated mice (Figures 5F and 5G). Additionally, immunohistochemistry and immunofluorescence demonstrated that p75^NTR+^ cells were upregulated and co-localized with B220^+^ splenic B cells in pristane-treated mice (Figures 5H and 5I). Importantly, pristane treatment led to an increase in the proportion of CD21^lo^ B cells in the spleen. In mice with B cell-specific p75^NTR^ deficiency, this increase was greater, along with a decrease in CD21 MFI in splenic B cells (Figures 5J–5M). These results further support the role of p75^NTR^ in regulating CD21^lo^ B cell expansion under autoimmune-like conditions.

### Upregulation of CD21^lo^-related transcriptomes in B cells lacking p75^NTR^ in mice challenged with pristane

**Figure 6:**
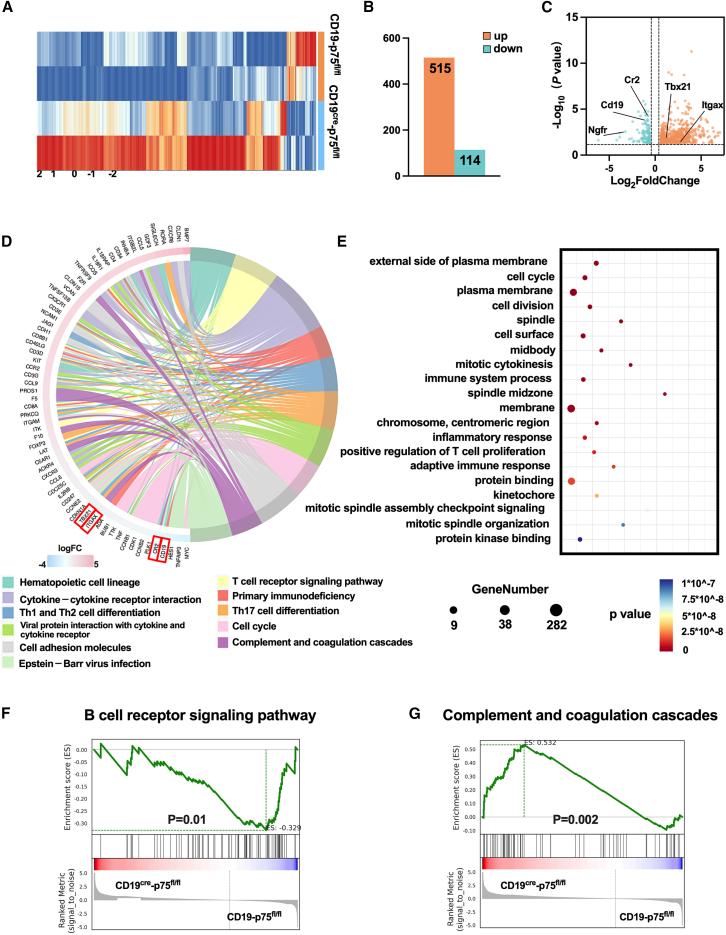
Upregulation of CD21^lo^-related transcriptomes in B cells lacking p75^NTR^ in mice challenged with pristane (A) Heatmap depicting differentially expressed transcripts in splenic B cells between CD19^cre^-p75^fl/fl^ mice and controls with pristane injection. Red indicates increased relative expression, and blue indicates decreased relative expression. (B) Statistical chart of DEGs. (C) The volcano plot shows differentially expressed genes. The red and green dots indicate upregulated and downregulated DEGs, respectively, with *p*-value <0.05. (D) Chord plot depicting the relationship between DEGs and KEGG pathways in splenic B cells in mice with pristane injection. (E) GO enrichment analysis of DEGs. (F and G) Enrichment plot from the B cell receptor signaling pathway and complement and coagulation upon GSEA with the DEGs obtained from the RNA-seq analysis in B220+ B cells.

Correspondingly, we analyzed the transcriptomic differences in B cells lacking p75^NTR^ in mice 4 months following pristane challenges. As shown, there were 659 DEGs in B cells between CD19^cre^-p75^fl/fl^ mice and the controls after being immunized with pristane, among which 515 genes were upregulated and 114 genes were downregulated (Figures 6A and 6B). In the DEGs, we found that *Cd19*, *Cr2*, and *Ngfr* expression decreased. The expression of *Tbx21* and *Itgax* (*Cd11c*), which were highly expressed in the CD21^lo^ B cell subtype were increased (Figure 6C). Chord workflow of KEGG enrichment revealed pathways involved in hematopoietic cell lineage, cytokine-cytokine receptor interaction, Th1 and Th2 cell differentiation, complement and coagulation cascades, and so on (Figure 6D). Gene ontology (GO) analysis indicated that the gene differences were enriched in immune system process, inflammatory response, and positive regulation of T cell proliferation, etc (Figure 6E). Concordant with this finding, GSEA showed inhibition of the B cell receptor signaling pathway and activation of the complement and coagulation cascades in splenic B cells in CD19^cre^-p75^fl/fl^ mice immunized with pristane when compared to control littermate (Figures 6F and 6G).

**Figure 7:**
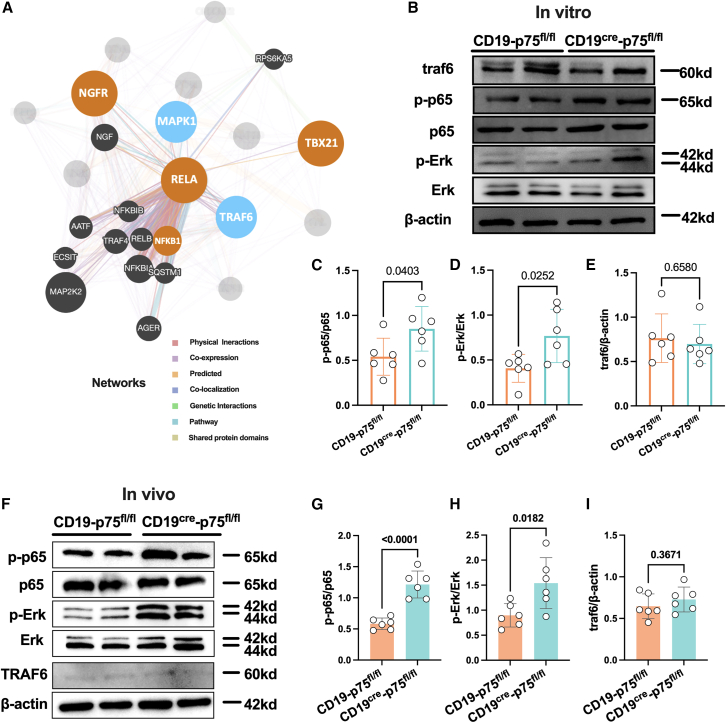
Deficiency of p75^NTR^ in B cells resulted in further elevation of phosphorylated-p65 and phosphorylated-Erk in splenic B cells following pristane challenge (A) A network diagram generated using GeneMANIA illustrates the protein and genetic interactions, pathways, and co-expression patterns involving *Ngfr* downstream and *Tbx21*. (B–E) The expression levels of p65, phosphorylated p65 (p-p65), Erk, phosphorylated ErK (*p*-Erk), traf6, and β-actin were analyzed in CD19^cre^-p75^fl/fl^ mice and control groups following CpG-B and R848 treatment for 24 h. (F–I) The expression of p65, p-p65, Erk, *p*-Erk, traf6, and β-actin was also evaluated in CD19^cre^-p75^fl/fl^ mice and control groups after pristane challenge. Horizontal bars represent the mean ± SEM of at least three independent experiments. Statistical analyses were performed using unpaired two-tailed Student’s *t* tests.

Finally, we used GeneMANIA to investigate the potential co-expression, genetic interactions, and pathways involving *Ngfr* downstream and *Tbx21*, aiming to explore the possible downstream signals responsible for p75^NTR^-induced expansion of CD21^lo^ B cell subsets. The gene set uploaded for analysis included *Nfkb1*, *Rela*, *Mapk1*, *Traf6*, *Ngfr*, and *Tbx21*, with the first three genes being well-established in pathways related to p75^NTR^ activity. As expected, the network revealed strong physical interactions, co-expression, and pathway involvement among *Ngfr*, *Rela*, *Nfkb1*, *Mapk1*, and *Traf6*. Notably, co-expression and predicted interactions were observed between *Rela* and *Tbx21*, suggesting a potential functional relationship (Figure 7A). Several previous studies have reported the regulatory role of p-p65 in driving Tbx21 activity in T cells.^35^ To further explore this, we examined downstream signaling changes in B cells following stimulation with TLR7/9. Stimulation with CpG-B and R848 significantly elevated the activity of p-p65, *p*-Erk, and Traf6 in B cells (Figure S5). In comparison to controls, p75^NTR^ knockout in B cells resulted in further upregulation of p-p65 and *p*-Erk, but not Traf6, when challenged with immune activation (Figures 7B–7E). Similarly, the pristane challenge led to increased levels of p-p65 and *p*-Erk in splenic B cells of CD19^cre^-p75^fl/fl^ mice (Figures 7F–7H), with no significant differences in Traf6 expression (Figure 7I). These results suggest that p-p65 may play a role in p75^NTR^-induced expansion of CD21^lo^ B cell subsets during TLR-mediated immune challenges.

## Discussion

B cells play a pivotal role in the modulation of immune responses. In this study, we demonstrated that murine B cells express low levels of p75^NTR^ in a steady state, but show upregulated expression following TLR stimulation *in vitro* and pristane immunization *in vivo*. Notably, we identified that the specific knockout of p75^NTR^ in B cells elevated the loss of CD21 expression, thereby increasing the proportion of a recently identified CD21^lo^ population of B cell subsets under autoimmune-like challenges. Transcriptomic analysis revealed a parallel elevation of CD21^lo^-driven transcriptomes, including Tbx21, and related enrichment of proinflammatory, antigen-presenting, and immune crosstalk pathways. Therefore, our study proposes that increased expression of p75^NTR^ in B cells restrains the development of CD21^lo^ subsets of B cells when facing immune challenges.

Despite the fact that p75^NTR^ belongs to the tumor necrosis factor receptor superfamily (TNFRSF), it performs multiple biological functions not shared with other TNFR members. Predominantly, it serves as a receptor for neurotrophic factors, usually by binding to nerve growth factor (NGF) or proBDNF, regulating neuronal apoptosis and pruning.^36^^,^^37^^,^^38^ Besides its well-documented role in the nervous system, researchers have demonstrated the expression and role of p75^NTR^ in immune systems, particularly in plasmacytoid dendritic cells (pDCs). Bandola et al. reported that p75^NTR^ is expressed in pDCs after TLR9 activation and modulates the disease progression of asthma in an interferon regulatory factor 3 and 7 dependent pathway.^11^ Additionally, p75^NTR^ expression increases in innate immune cells during neuronal infection,^39^ and knockout of p75^NTR^ mitigates neuroinflammation damage.^7^ Similar p75^NTR^ expression in myeloid cells has also been proven.^40^^,^^41^ A recent study using *Ngfr* full-knockout mice showed that loss of *Ngfr* leads to spontaneous activation of follicular dendritic cells, upregulation of B-cell activation markers, generation of autoreactive clones, and increased autoantibody production.^16^

Our team has explored the immune regulatory role of p75^NTR^, supporting its expression in not only monocytes/macrophages^13^^,^^42^ but also in B and T lymphocytes.^15^^,^^43^ We recently identified that the precursor of brain-derived neurotrophic factor elevated in antibody-secreting B cell subsets drives the development of systemic lupus erythematosus in a p75^NTR^-dependent manner,^15^ highlighting an immune-modulating role of proBDNF-p75^NTR^ signaling in B cells. In this manuscript, we further identified a close relationship between the elevation of p75^NTR^ in response to autoimmune-like challenges and the suppression of CD21 expression in B lymphocytes. Thus, our findings extend and validate the potential role of p75^NTR^ in B lymphocytes in immune regulation.

Although the definition of CD21^lo^ B cells remains unclear, these cells have garnered particular interest in recent years due to their increased frequency with age and during chronic infections and autoimmune inflammatory conditions.^22^^,^^28^^,^^44^^,^^45^ While much attention has been given to understanding the characteristics of these transitional B cell subsets, studies on their regulation are limited. CD21^lo^ B cells are mature B cells, as evidenced by their lack of CD93. They are positive for both B220 and CD19 but lack canonical follicular, marginal zone, or B1 B-cell markers CD23, CD21, and CD43, respectively. CD21^lo^ B cells express exceptionally high levels of co-stimulatory molecules such as CD80 and CD86^34^ and the type I transmembrane protein CD11c, which is highly expressed in some myeloid cells.^24^ These cells are characterized by a T-bet-driven transcriptional program, robust responsiveness to TLR7 and TLR9 ligands, and a propensity for IgG2a/c production.^23^^,^^46^ Additionally, CD21^lo^ B cells can transform into long-lived plasma cells by activating the Blimp-1 program and expressing plasma cell markers.^19^

In the present study, we reported a link between increased p75^NTR^ expression and the development of CD21^lo^ B cell subsets induced by autoimmune signals. It has been reported that B cell activation induces CD21 shedding.^47^ Our results align with these previous findings, indicating that CD21 expression is downregulated after both TLR7 and TLR9 activation *in vitro* as well as pristane stimulation *in vivo*. Under these conditions, p75^NTR^ expression was significantly induced in B cells, while p75^NTR^ knockdown further promoted the population of CD21^lo^ subsets. The upregulation of CD21^lo^ B cell-driven genes, including *Tbx21* and *Itgax*, and alterations in related signaling pathways in p75^NTR^-deficient B cells from RNA-seq data support a regulatory role for p75^NTR^ in CD21 expression in B lymphocytes. Upon TLR engagement, p75^NTR^ expression increases and acts to dampen downstream proliferative signals—potentially by attenuating NF-κB and/or ErK pathway activity—to prevent excessive expansion of CD21^lo^ B cells and uncontrolled inflammation. In the p75^NTR^-knockout setting, this control is lost, so TLR signals drive even greater CD21^lo^ B-cell proliferation. Thus, TLR-induced p75^NTR^ serves to limit CD21^lo^ expansion, reconciling our observations of both inducible up-regulation and enhanced proliferation in the absence of p75^NTR^. This evidence indicates an intrinsic p75^NTR^-dependent inhibitory pathway that suppresses the overproduction of CD21^lo^ subsets in B cells facing immune disorders, especially those related to TLR signals. Hence, our findings contribute to an understanding of the intricate molecular mechanisms associated with p75^NTR^ in regulating B cell development amidst immune dysregulations.

Multiple functional downstream pathways are involved in p75^NTR^’s function, which vary by cell type and disease. Of these, NRAGE, NADE, and NRIF have been associated with the induction of apoptosis, while FAP-1, RIP2, and TRAF6 appear to promote cellular survival.^11^ A recent gene network interaction analysis identified 10 highly interconnected genes with p75^NTR^: *Stat1*, *Stat3*, *Sp1*, *Jun*, *Egfr*, *Traf6*, *Jak2*, *Nras*, *Tp53*, and *Mdm2*.^48^ Therefore, identifying the signaling cascades activated by p75^NTR^ is complex and challenging, especially when linking various p75^NTR^ binding proteins to specific p75^NTR^-dependent functions. In our present study, we observed increased expression of p-p65, *p*-ErK, and Traf6 in B cells after TLR stimulation *in vitro*. The deficiency of p75^NTR^ further elevated the expression of p-p65 and *p*-ErK but not Traf6. This implies that activation of p75^NTR^ in B cells may modulate the CD21^lo^ B cell subpopulation through downstream signals involving p-p65 and *p*-ErK. A report from Bandoła et al. studying pDCs indicated that the deficiency of p75^NTR^ resulted in lower levels of phosphorylation of IRF3, IRF7, IKKα/β, and c-Jun, but not MyD88, IRAK4, TRAF6, PI3K, etc., when compared to control.^11^ Thus, it is possible that TRAF6 is not crucial for the immune regulatory role of p75^NTR^. However, we must acknowledge that the crucial question remains unanswered: the precise mechanism by which p75^NTR^ signaling limits the accumulation of CD21^lo^ subsets and B cell hyperactivation under immune disorder conditions remains unanswered.

Additionally, different ligands activate different downstream pathways of p75^NTR^. The high-affinity ligand for p75^NTR^ is proBDNF, but mature BDNF also binds to it and elicits biological functions. Neurotrophic factors and their receptors are differentially expressed throughout B lymphocyte development, such as in human-activated B cells, memory B cells, and mouse spleen and bone marrow B cells.^49^ In our previous study, we found that immune cells, including myeloid cells and lymphocytes, have the potential to secrete proBDNF and modulate immune responses. Hence, it will be interesting to further investigate whether proBDNF from immune cells acts as an autocrine or paracrine regulatory mechanism involved in regulating p75^NTR^ in CD21^lo^ B cell subsets. Given the high affinity of proBDNF in enhancing p75^NTR^ signaling, strategies that modulate proBDNF levels or enhance its interaction with p75^NTR^ may represent promising avenues for intervention. Such approaches—including the administration of exogenous protease-resistant proBDNF or small molecules that promote p75^NTR^ activation—could help restore immune homeostasis in patients with dysregulated B cell responses.

In summary, the central finding of our present study provides evidence that the functional expression of p75^NTR^ by B cells plays a critical role in restraining CD21^lo^ B cell subsets within the context of autoimmune pathology. The absence of p75^NTR^ in B cells led to an increased ratio of CD21^lo^ B cell subsets, accompanied by overactivation of these cells and significant changes in their transcriptomic profile. Our results provide insights into the role of p75^NTR^ in the regulation of B cell activation and function and in preserving immune homeostasis amidst disorders.

### Limitations of the study

There are several limitations in the present study. While our study primarily focused on the CD21^lo^ B cell subset due to its established relevance in autoimmune responses, we acknowledge that p75^NTR^ deficiency may have broader effects on other B cell populations. A detailed characterization of additional B cell subsets was not performed in this study and remains an important avenue for future investigation. In addition, we acknowledge that we did not assess downstream autoimmune parameters such as autoantibody levels, inflammatory cytokine profiles, or kidney pathology in the pristane-induced lupus model. These represent important limitations of the current study. Future investigations will determine whether the expansion of CD21^lo^ B cells in the absence of p75^NTR^ contributes functionally to autoimmune disease progression. Furthermore, although our findings suggest that p75^NTR^ modulates TLR7/9-driven responses in B cells, the downstream signaling mechanisms remain incompletely defined. Future studies using pharmacological inhibitors and genetic perturbations of key signaling intermediates will be essential to establish a direct mechanistic link.

## Resource availability

### Lead contact

All requests for reagents and resources should be directed to the lead contact, Ru-Ping Dai (xyeyyrupingdai@csu.edu.cn).

### Materials availability

This study did not generate new unique reagents.

### Data and code availability

RNA-sequencing data of this work have been deposited at Sequence Read Archive (SRA) data repository and are publicly available as of the date of publication. Accession numbers (SRA: PRJNA1182849) are listed in the key resources table. This paper does not report original code. Any additional information required to reanalyze the data reported in this paper is available from the lead contact upon request.

## Acknowledgments

This study was supported by the 10.13039/501100001809National Natural Science Foundation of China (NSFC 82371292 to RD, 82101285 to CL, 82102284 to WS, 82271379 to ZH), Science and Technology Innovation Program of Hunan Province (2021RC4015 to RD), Science Foundation of Hunan province for Distinguished Young Scholars (2023JJ10088 to ZLH) and the Fundamental Research Funds for the Central Universities of 10.13039/501100002822Central South University (2023XQLH146 to AHZ).

## Author contributions

Conceptualization: C.L., W.-Y.S., and R.-P.D.; methodology: C.L., A.-H.Z., W.-Y.S., R.-Y.L., and R.-P.D.; investigation: C.L., A.-H.Z., and W.Y.S.; writing – original draft: C.L., A.-H.Z., W.-Y.S.; writing – review and editing: C.L., A.-H.Z., W.-Y.S., R.-Y.L., Z.-L.H., and R.-P.D.; funding acquisition: C.L., W.-Y.S., and R.P.D.; and supervision: C.L., W.-Y.S., Z.-L.H., and R.-P.D.

## Declaration of interests

The authors declare no competing interests.

## STAR★Methods

### Key resources table

**Table:** 

REAGENT or RESOURCE	SOURCE	IDENTIFIER
**Antibodies**		

PerCP/Cyanine5.5 anti-mouse CD45	Biolegend	Cat# 157208; RRID:AB_2860728
APC anti-mouse/human CD45R/B220	Biolegend	Cat# 103212; RRID:AB_312997
PE anti-mouse CD21/CD35 (CR2/CR1)	Biolegend	Cat# 123409; RRID:AB_940411
FITC anti-mouse CD21/CD35(CR2/CR1)	Biolegend	Cat# 123407; RRID:AB_940403
APC/Cyanine7 anti-mouse CD23	Biolegend	Cat# 101630; RRID:AB_2571987
PE-*anti*-human/mouse CD271	Thermo Fisher	Cat# 12-9400-41; RRID:AB_2572709
FITC-*anti*-mouse CD86	Biolegend	Cat# 159220; RRID:AB_3106043
BV421-*anti*-mouse CD3	BD	Cat# 740014; RRID:AB_2739786
PE/Cyanine7 anti-mouse/human CD44	Biolegend	Cat# 103030; RRID:AB_830787
p75NTR Rabbit mAb	CST	Cat# 8238; RRID:AB_10839265
TRAF6 Rabbit mAb	CST	Cat# 8028; RRID:AB_10858223
Sortilin Polyclonal antibody	Proteintech	Cat# 12369-1-AP
NF-κB p65 Rabbit mAb	CST	Cat# 8242; RRID:AB_10859369
Phospho-NF-κB p65 Rabbit mAb	CST	Cat# 3033; RRID:AB_331284
p44/42 MAPK (Erk1/2) Rabbit mAb	CST	Cat# 4695; RRID:AB_390779
Phospho-p44/42 MAPK (Erk1/2) Rabbit mAb	CST	Cat# 4370; RRID:AB_2315112
HRP-Conjugated Beta Actin Monoclonal Antibody	Proteintech	Cat# HRP-60008
GAPDH Polyclonal antibody	Proteintech	Cat# 10494-1-AP
HRP-conjugated Goat Anti-Mouse IgG(H + L)	Proteintech	Cat# SA00001-1
HRP-conjugated Goat Anti-Rabbit IgG(H + L)	Proteintech	Cat# SA00001-2
Biotin-conjugated Goat Anti-Rabbit IgG(H + L)	Proteintech	Cat# SA00004-2

**Chemicals, peptides, and recombinant proteins**		

CD45R(B220) microbeads, mouse	MiltenyiBiotec	Cat# 130-049-501
Pristane	Sigma-Aldrich	Cat# P2870
CpG-B (ODN 1826)	InvivoGen	Cat# tlrl-1826
R848	InvivoGen	Cat# tlrl-r848
Fixation buffer	Invitrogen	Cat# 00-8222-49
Compensation Beads	Invitrogen	Cat# 01-3333-42
Red cells lysis buffer	Biosharp	Cat# BL503B
Tween 20	Biosharp	Cat# BS100
Triton X-100	Biosharp	Cat# BS084
1X PBS (without Ca^2+^)	Biosharp	Cat# BL302A
Ficoll	GE Health	Cat# 17-1440-02
DMEM basic (1X)	Gibco	Cat# C11995500BT
Fetal bovine serum	Gibco	Cat# 10091-148
Streptomycin/penicillin	Gibco	Cat# 15140-12
L-Glutamine	Gibco	Cat# A2916801
TRIzol	Ambion	Cat# 15596076
SYBR qPCR SuperMix	Novoprotein	Cat# E096
SDS-PAGE	Epizyme	Cat# PG112
RIPA lysis buffer	Beyotime	Cat# P0013C
5X SDS-PAGE Sample Loading Buffer	Beyotime	Cat# P0015
PageRuler Prestained Protein ladder	Thermo Fisher	Cat# 26616
DAPI	Southern Biotech	Cat# 0100-20
1X TAE	Tsingke	Cat# TSG001
100 bp DNA Ladder	Tsingke	Cat# TSJ100-100
Agarose	Tsingke	Cat# TSJ001
Immobilon ECL Ultra Western HRP	Millipore	Cat# WBULS0100

**Critical commercial assays**		

BCA Protein Assay Kit	Bioss	Cat# C05-02001
LEGENDplex™ Carboxyl Beads A4	Biolegend	Cat# 740167
DAB color development kit	ZSBIO	Cat# ZLI-9019
ABC HRP Kit	Vector Labs	Cat# PK-6100
RevertAid First Strand cDNA Synthesis Kit	Thermo	Cat# K1622
Zombie NIR™ Fixable Viability Kit	Biolegend	Cat# 423105

**Deposited data**		

Mice splenic B cells RNA-sequencing data	This paper	SRA: PRJNA1182849

**Experimental models: Cell lines**		

Mice: splenic lymphocytes	N/A	N/A
Mice: splenic B cells	N/A	N/A

**Experimental models: Organisms/strains**		

C57BL6/J	Hunan Silaikejingda Experimental Animal	N/A
P75^fl/fl^ mice	Beijing Viewsolid Biotech	N/A
CD19-Cre	Beijing Viewsolid Biotech	N/A

**Software and algorithms**		

PRISM	GraphPad	Version 10
Flow Jo	Flowjo	Version 10
ImageJ	Rawak Software	https://imagej.nih.gov/ij/

**Other**		

BD Trucount tubes	BD	Cat# 340334
PVDF transfer membranes	MerckMillipore	Cat# ISEQ00010
70 μm-mesh Nylon cell strainer	Biosharp	Cat# BS-70-CS

### Experimental model and study participant details

#### Mice

P75^fl/fl^ mice (on C57BL/6J background), provided by Beijing Viewsolid Biotech Co. Ltd, were generated using *loxP* system targeting exon 3 of the *Ngfr* gene. The p75^fl/fl^ mice were crossed with CD19-Cre transgenic mice (B6 genetic background, Beijing Viewsolid Biotech Co. Ltd) to create CD19^cre^-p75^fl/fl^ and wild-type (p75^fl/fl^) mice. C57BL/6J mice were purchased from SLAC Laboratory Animal Co. Ltd. All mice were housed in individually ventilated cages in a specific pathogen-free environment and under standard conditions (20°C–26°C, 40–60% humidity, 12:12 h light-dark cycle) with *ad libitum* access to food and water at Central South University Animal Services (Changsha, China). Animal procedures were approved by the Animal Research Ethics Committee of Xiangya Hospital, Central South University (no. 2020147, Changsha, China) and conformed to the National Institutes of Health Guide for the Care and Use of Laboratory Animals. The pristane-induced lupus model utilized 6-week-old female mice. All cellular experiments were performed using adult mice (8–12 weeks old) without sex restriction.

#### Primary cell cultures

B220^+^ splenocytes were magnetically isolated from the spleens of 8–12 week old C57BL/6J mice, or from CD19^cre^-p75fl/fl mice and CD19-p75^fl/fl^ littermate controls. Isolated cells were resuspended in DMDM complete medium (10% fetal bovine serum, 1% streptomycin/penicillin) in 37°C and 5% CO_2_.

### Method details

#### Pristane immunization

A pristane-immunized mouse model was induced by injecting 0.5 mL of pristane intraperitoneally (i.p.) into 6-week-old female mice; PBS i.p. injection was used as a control. Eighteen and twenty-two weeks post-immunization, the mice were sacrificed for further experiments.

#### Western blot

For protein extraction, tissues, and cells were lysed on ice using RIPA buffer supplemented with a protease inhibitor and phosphatase inhibitor cocktail. Protein concentration of lysates was determined using the BCA Protein Assay Kit according to the manufacturer’s instructions. Cell lysates were boiled for 8 min at 100°C with SDS, and subjected to 10% SDS–PAGE, and then transferred to PVDF membranes. After blotting, the membrane was blocked in 5% non-fat dry milk diluted in TBS-tween (TBST 0.5%) for 1 h to prevent unspecific antibody binding. Then, the membrane was incubated with anti-p75^NTR^ (1:1000), anti-Traf6 (1:1000), anti-p65 (1:1000), anti-p-p65 (1:1000), anti-Erk (1:1000), anti-*p*-Erk (1:1000), anti-β-actin (1:5000), or anti-GAPDH (1:5000) overnight at 4°C. Subsequently, the membrane was thoroughly washed and incubated with the horseradish peroxidase (HRP)-conjugated secondary antibody specific to the species of the primary antibodies (1:5000) in 5% dry milk. ECL substrate was used for the development. Protein bands were visualized with the western blotting detection system (CLINX, Shanghai, China). Gray value analysis was done by ImageJ (v.1.50g, NIH) software.

#### Cell isolation and *in vitro* culture

The spleen was collected to prepare a single-cell suspension for the isolation of mice splenic B cells. Splenic lymphocytes were isolated through Ficoll density gradient centrifugation. Cells were then washed and resuspended in PBS. According to the manufacturer’s instructions, B220^+^ B cells were isolated from splenic lymphocytes using mouse CD45R(B220) microbeads. The purity of enriched B220^+^ B cells was determined by flow cytometry and was higher than 95%. Cells were cultured in complete Dulbecco’s modified Eagle’s medium with 10% fetal bovine serum, 1% streptomycin/penicillin in 96-well round-bottom plates in which each well contained 3 × 10^6^ cells in 200 μL of medium at 37°C in a humidified atmosphere of 20% O_2_ and 5% CO_2_. The cells were stimulated with R848 (200 ng/mL) or CpG-B (0.25 μmol) for activation. After 24 h, the cells were collected for flow cytometry, immunofluorescence, quantitative PCR and RNA-seq analysis.

#### Flow cytometry

To determine B cell subsets and p75^NTR^ expression, splenic lymphocytes or cultured cells were harvested and washed with phosphate-buffered saline (PBS). Cells were then incubated with fixable viability dyes for 10 min at room temperature. Then cells were washed with PBS (containing 1% BSA), and then stained with antibodies against different cell surface markers by incubation for 30 min at 4°C. Stained cells were washed and then read on a flow cytometer (Cytek, Fremont, California, USA). Data were analyzed using FlowJo V.10.4 software.

#### Cytometric bead-based immunoassays of multiple soluble analytes

Culture supernatants were collected and stored at −80°C for further detection. Cytokine levels were measured using a LEGENDplex multianalyte flow assay kit according to the manufacturer’s protocol, and the concentration of cytokines was determined by using LEGENDplex software (BioLegend).

#### Tissue immunohistochemistry and immunofluorescence

Spleens were fixed in 4% paraformaldehyde, and sectioned (4 μm) for immunohistochemical (IHC) analysis. Sections were deparaffinized in xylene and then rehydrated with graded alcohols. Antigen retrieval was done by microwave boiling in 10 mM citrate buffer for 20 min. Endogenous peroxidase was blocked using 3% H_2_O_2_ in methanol for 15 min. The slides were then washed with PBS and blocked using 10% BSA with 0.5% Triton X-100 in PBS for 1 h. The sections were incubated with rabbit anti-p75^NTR^ antibody (1:500) at 4°C overnight, followed by incubation with biotin goat anti-Rabbit IgG (H + L) for 1 h. The slides were washed three times in PBS and incubated with the ABC HRP Kit. Staining was developed using DAB staining. The sections were dehydrated with increasing grades of alcohol and xylene, and then sealed with neutral resin. For immunofluorescence (IFC) staining, slides were incubated with rat anti-CD45R antibody (1:500) and rabbit anti-p75^NTR^ antibody (1:500) at 4°C overnight, followed by incubation with Alexa Fluor 647 Donkey anti-Rat IgG and Alexa Fluor 488 goat anti-Rabbit IgG (H + L) for 1 h. After rinsing, slides were further stained with 4′,6-diamidino-2-phenylindole (DAPI). Images were collected by a scanning microscope (Pannoramic DESK, P-MIDI, P250, P1000) and analyzed using Case Viewer software.

#### Cell immunofluorescence

To assess the expression of p75^NTR^ on B cells, 1×10^5^ splenic B cells were collected after R848 (200 ng/mL), CpG-B (0.25 μmol), or PBS control treatment, and fixed in 4% paraformaldehyde at room temperature for 10 min. Subsequently, the cells were washed with PBS and blocked using 5% BSA with 0.3% Triton X-100 in PBS for 30 min. The sections were then incubated with rabbit anti-p75^NTR^ antibody (1:1600) at room temperature for 2 h, followed by incubation with Alexa Fluor 488 goat anti-Rabbit IgG (H + L) for 1 h. The cell suspension was transferred onto an adhesive slide and allowed to dry before being further stained with DAPI. Images were collected by a scanning microscope (Pannoramic DESK, P-MIDI, P250, P1000).

#### RNA-seq analysis

Total RNA of splenic B cells from cell culture and mice was extracted with TRIzol, and subjected to RNA-seq analysis. RNA-seq was performed by OE (Shanghai, China) Biotechnology using the Illumina NovaSeq 6000 platform. Raw reads of fastq format were first processed through in-house perl scripts, and all the downstream analyses were based on clean data of high quality. Transcripts with a *P*-adjust <0.05 and fold change≥2 were assigned as differentially expressed.

#### Quantitative PCR

Total RNA from splenic B cells was extracted with TRIzol reagent and was reverse transcribed using revertAid first-strand complementary DNA (cDNA) synthesis kits. Diluted cDNAs were used as templates for qPCR with Fast SYBR Green Master Mix in the CFX96 Touch Deep-Well Real-Time PCR Detection System (Bio-Rad, Hercules, CA, USA). The primer sequences used are shown in the Table below.

**Table:** 

Gene	Forword Primer (5′-3′)	Reverse Primer (3′-5′)
*Tbx21*	TCAACCAGCACCAGACAGAG	AAACATCCTGTAATGGCTTGTG
*Zbp1*	GACGACAGCCAAAGAAGTGA	GAGCTATGTCTTGGCCTTCC
*Ifng*	CGGCACAGTCATTGAAAGCCTA	GTTGCTGATGGCCTGATTGTC
*Il12b*	TGGTTTGCCATCGTTTTGCTG	ACAGGTGAGGTTCACTGTTTCT
*Tgtp1*	TGGGACCACTAACTTCACACC	GGCCAGTTGTGCATCATTTTC
*Aicda*	GCCACCTTCGCAACAAGTCT	CCGGGCACAGTCATAGCAC
*Jchain*	TGACGACGAAGCGACCATTC	TTCAAAGGGACAACAATTCGGA
*Egln3*	AGGCAATGGTGGCTTGCTATC	GCGTCCCAATTCTTATTCAGGT
*Gapdh*	TGTGTCCGTCGTGGATCTGA	TTGCTGTTGAAGTCGCAGGAG

### Quantification and statistical analysis

The significance of the differences between more than two groups was evaluated using ANOVA followed by Sidak’s multiple comparison test. Comparisons between two conditions were analyzed by unpaired 2-tailed Student’s t test. Statistical analyses were performed with Prism software (GraphPad). All data are presented as the mean ± SEM. *p* < 0.05 was considered significant. The statistical details of experiments can be found in the figure legends.

## Supplemental information

Document S1. Figures S1–S5

Data S1. Original Western blot data
