# Supplementary material for: P75NTR activation limits CD21lo B cell subsets expansion in response to autoimmune-inducing challenges
Source: iScience. 2025 Jul 3;28(8):113055. doi: 10.1016/j.isci.2025.113055 (PMC12303049; doi:10.1016/j.isci.2025.113055)
Supplement: Document S1. Figures S1–S5 [file mmc1.pdf]

iScience, Volume *28*

Supplemental information

P75^{NTR} activation limits CD21^{lo} B cell subsets expansion in response to autoimmune-inducing challenges

Cong Luo, An-Hui Zha, Ru-Yi Luo, Zhao-Lan Hu, Wei-Yun Shen, and Ru-Ping Dai

Supplementary figures

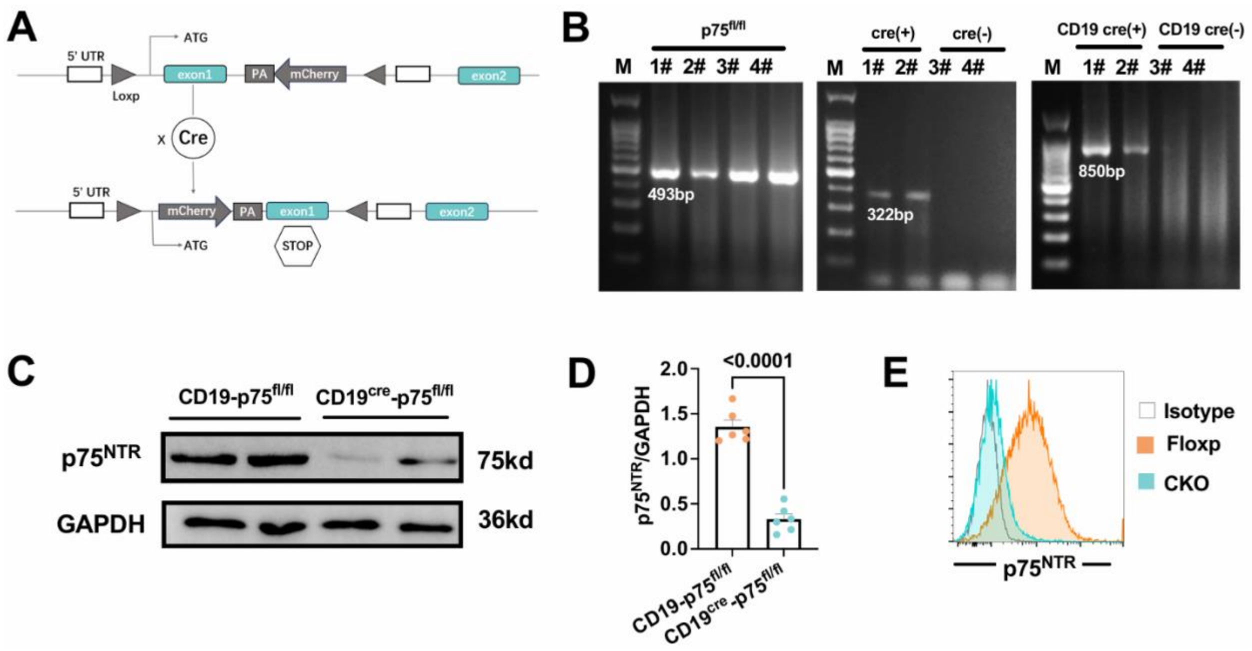

Fig. S1 Genotype identification of CD19 conditioning knockout of p75^{NTR} (CD19^{cre}-p75$^{fl/fl}$) mice. (A) The p75 mutant gene (p75$^{fl/fl}$) and inversion strategy were used to generate CD19^{cre}-p75$^{fl/fl}$ mice. (B) Identification of B cell-specific p75^{NTR} conditional knockout mice by 2% agarose gel electrophoresis analysis of PCR-amplified products from mouse tail. The 493bp PCR product band corresponds to p75$^{fl/fl}$ mice. The 322bp PCR product band indicated successful cre-mediated recombination. The 850bp PCR product band was observed in CD19^{cre}-p75$^{fl/fl}$ transgenic mice, and no band was observed in control littermates. (C-D) Confirmation of the p75^{NTR} knockout effect in CD19^{cre}-p75$^{fl/fl}$ mice by western blotting. (E) Confirmation of the p75^{NTR} knockout effect in CD19^{cre}-p75$^{fl/fl}$ mice by flow cytometry. Horizontal bars represent the mean ± SEM of at least three independent experiments. Statistical analyses were performed using unpaired two-tailed Student's t-tests.

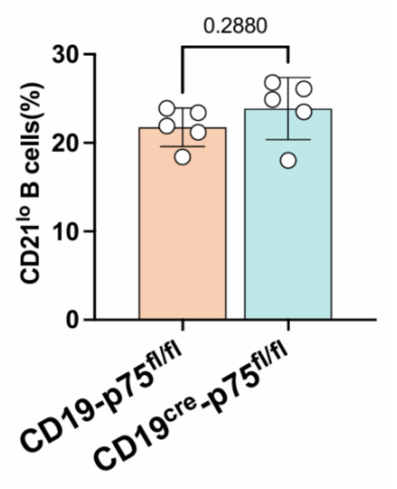

Fig. S2. No difference was found in $CD21^{lo}$ B cells between CD19-$p75^{fl/fl}$ mice and $CD19^{cre}$-$p75^{fl/fl}$ mice in baseline conditions. Horizontal bars represent the mean ± SEM. Statistical analyses were performed using unpaired two-tailed Student's t-tests.

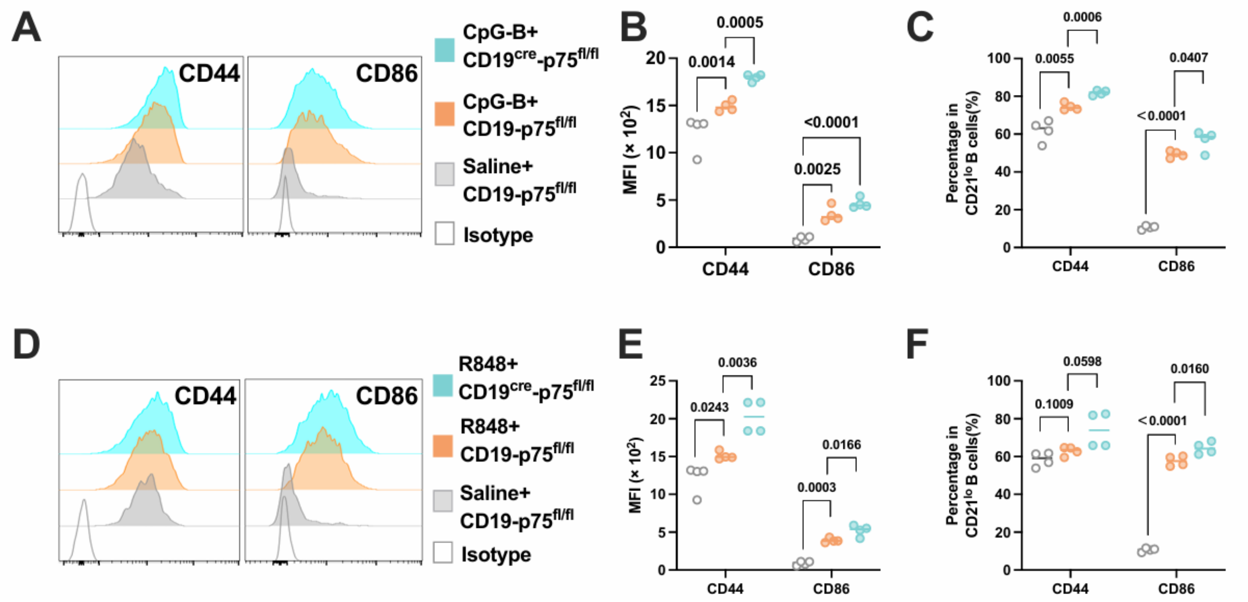

Fig. S3 p75^{NTR} deficiency in B cells increased the expression of CD44 and CD86 in CD21^{lo} B cell subsets. B220^{+} splenic B cells from CD19^{cre}-p75$^{fl/fl}$ mice or their CD19-p75$^{fl/fl}$ control were sorted with magnetic beads and then cultured with CpG-B (0.25 μmol) or R848 (200 ng/mL) for 24 h. (A-C) Representative flow cytometry histograms and statistical analysis showing CD44 and CD86 MFI (A-B), CD44, and CD86 positive cells (C) in CD21^{lo} B cell subsets after CpG-B treatment. (D-F) Representative flow cytometry histograms and statistical analysis showing CD44 and CD86 MFI (D-E), CD44 and CD86 positive cells (F) in CD21^{lo} B cell subsets after R848 treatment. Horizontal bars represent the mean ± SEM. Statistical analyses were performed using unpaired two-tailed Student's t-tests.

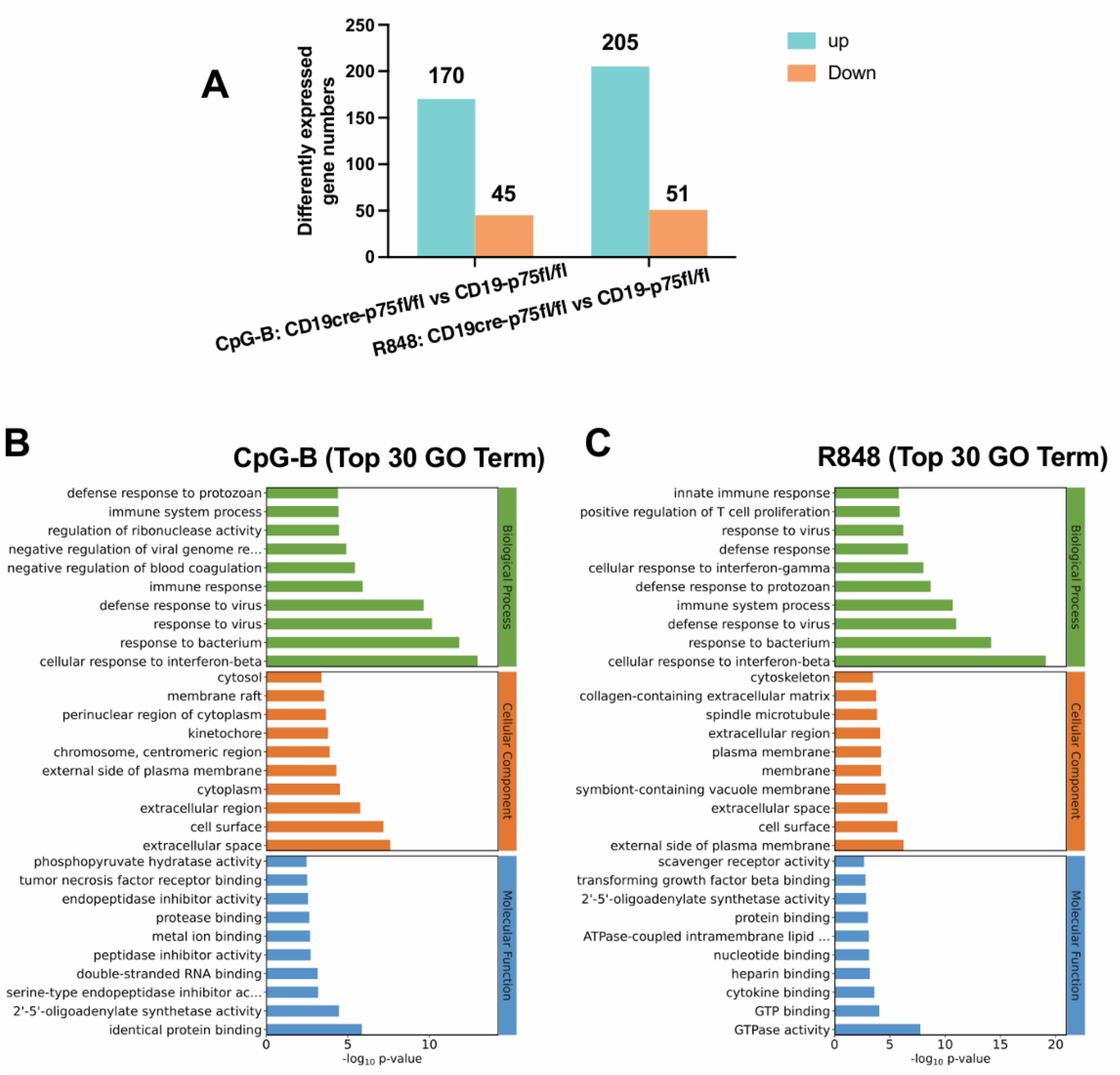

Fig.S4 GO enrichment analysis of DEGs. (A) Statistical chart of DEGs between CpG-B-stimulated and R848-stimulated differences in lacking $p75^{NTR}$ in B cells. (B) Top 30 GO terms for CpG-B treatment. (C) Top 30 GO terms for R848 treatment.

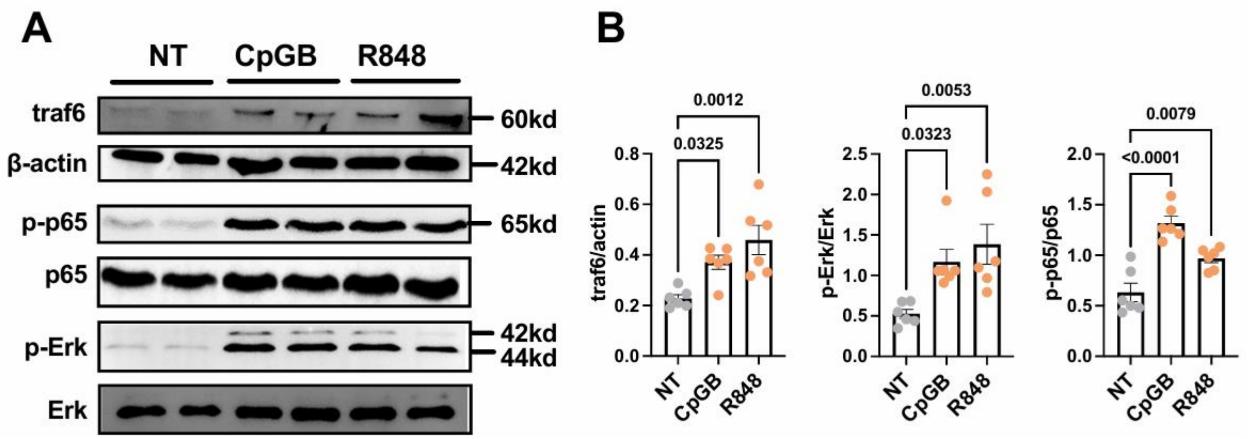

Fig.S5 Elevated p75^{NTR}-related molecules and signaling molecules in splenic B cells after TLR-activation. (A-B) Western blot images and statistical analysis of p75^{NTR}-related molecules, NF-κB, and ErK signal expression in splenic B cells after TLR-mediated activation. Horizontal bars represent the mean ± SEM of at least three independent experiments. Statistical analyses were performed using one-way ANOVA tests
