# Supplementary figures and images for: P75NTR activation limits CD21lo B cell subsets expansion in response to autoimmune-inducing challenges
Source: iScience. 2025 Jul 3;28(8):113055. doi: 10.1016/j.isci.2025.113055 (PMC12303049; doi:10.1016/j.isci.2025.113055)

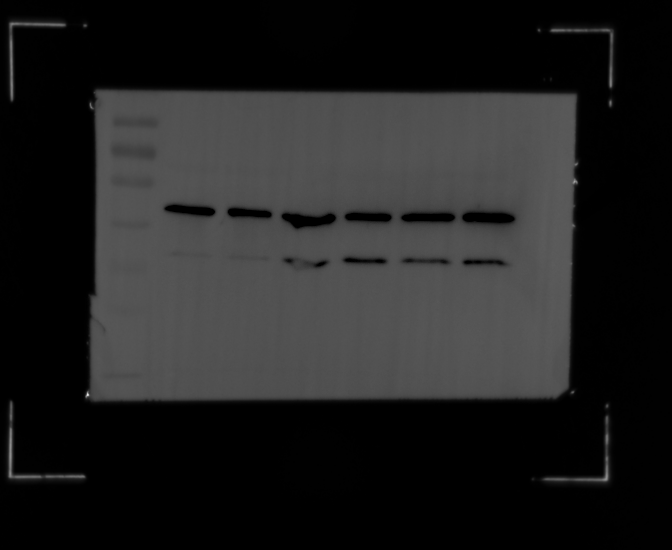

Supplement: Data S1. Original Western blot data [file mmc2.zip › WB原始图/iScience Fig1F-actin.tif]

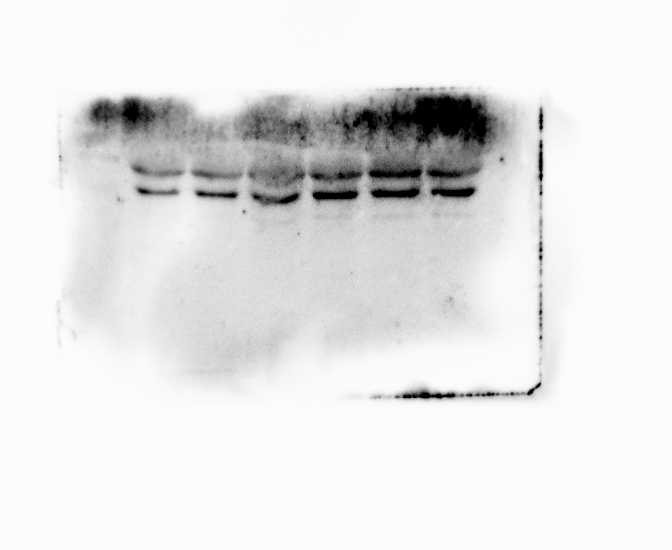

Supplement: Data S1. Original Western blot data [file mmc2.zip › WB原始图/iScience Fig1F-p75NTR.tif]

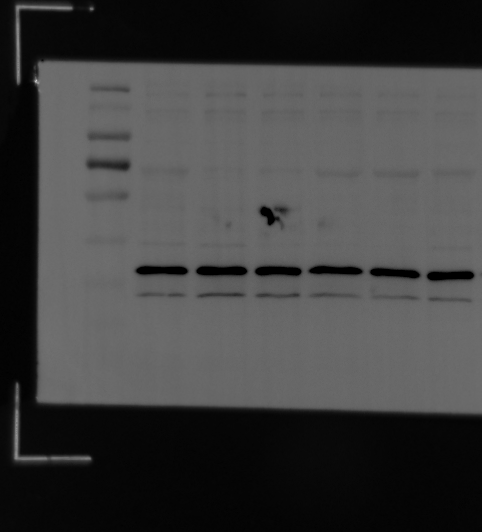

Supplement: Data S1. Original Western blot data [file mmc2.zip › WB原始图/iScience Fig5F-GAPDH.tif]

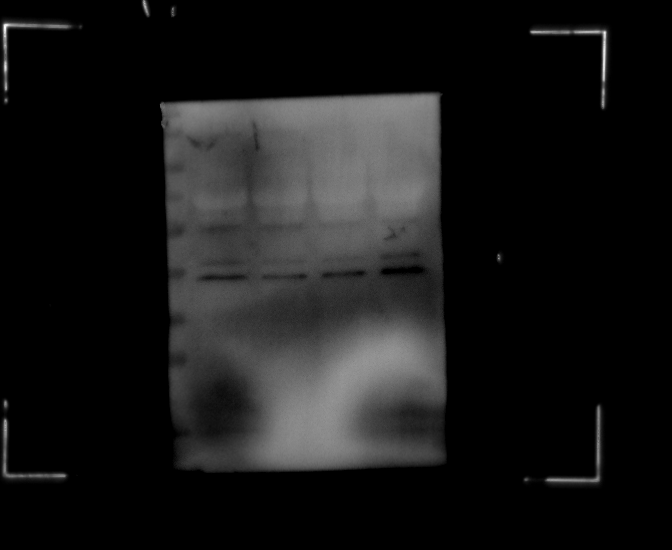

Supplement: Data S1. Original Western blot data [file mmc2.zip › WB原始图/iScience Fig7B-p-Erk.tif]

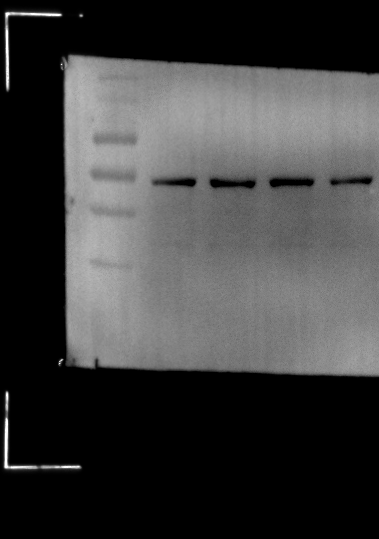

Supplement: Data S1. Original Western blot data [file mmc2.zip › WB原始图/iScience Fig7B-p-p65.tif]

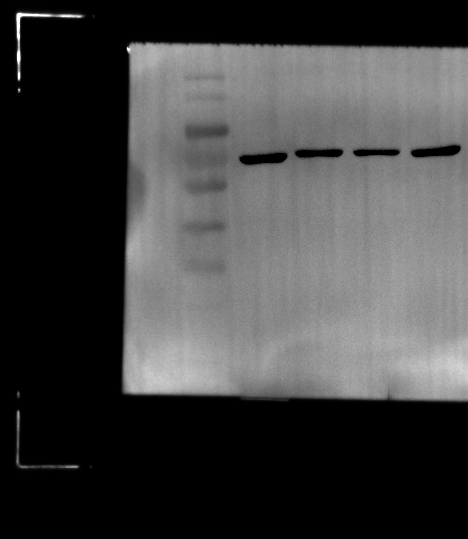

Supplement: Data S1. Original Western blot data [file mmc2.zip › WB原始图/iScience Fig7B-p65.tif]

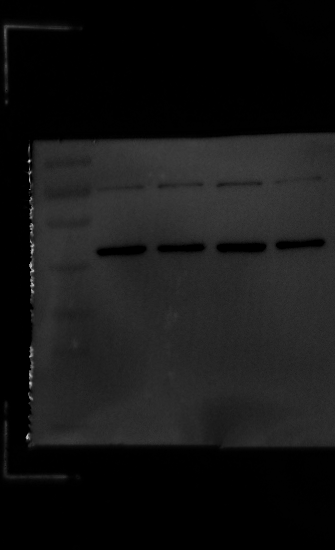

Supplement: Data S1. Original Western blot data [file mmc2.zip › WB原始图/iScience Fig7B-β-actin.tif]

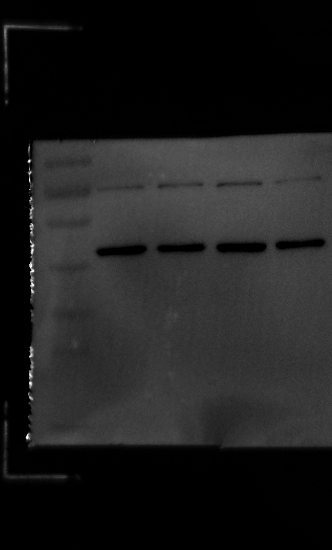

Supplement: Data S1. Original Western blot data [file mmc2.zip › WB原始图/iScience Fig7F-β-actin.tif]

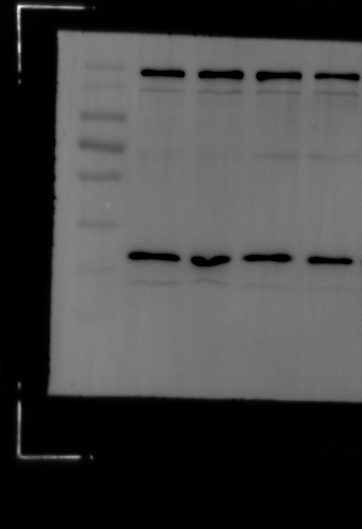

Supplement: Data S1. Original Western blot data [file mmc2.zip › WB原始图/iScience FigS1G-GAPDH.tif]

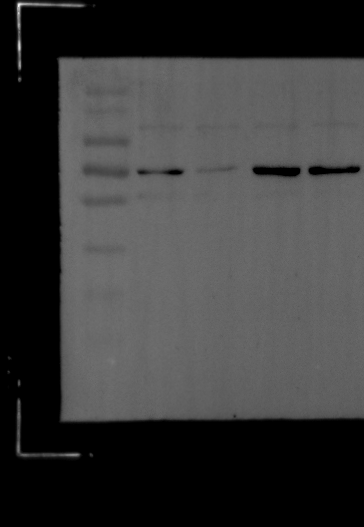

Supplement: Data S1. Original Western blot data [file mmc2.zip › WB原始图/iScience FigS1G-P75NTR.tif]

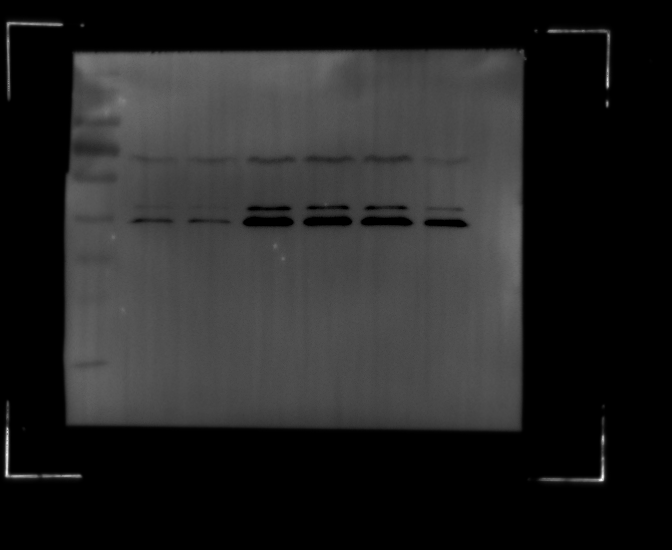

Supplement: Data S1. Original Western blot data [file mmc2.zip › WB原始图/iScience FigS5A-P-Erk.tif]

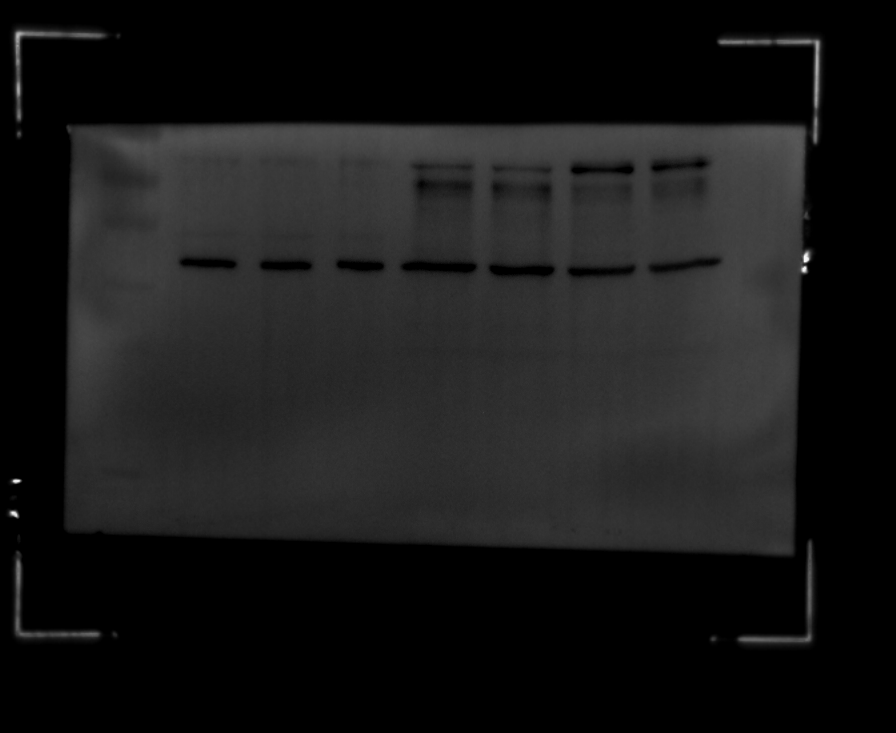

Supplement: Data S1. Original Western blot data [file mmc2.zip › WB原始图/iSicence Fig5F-p75NTR.tif]

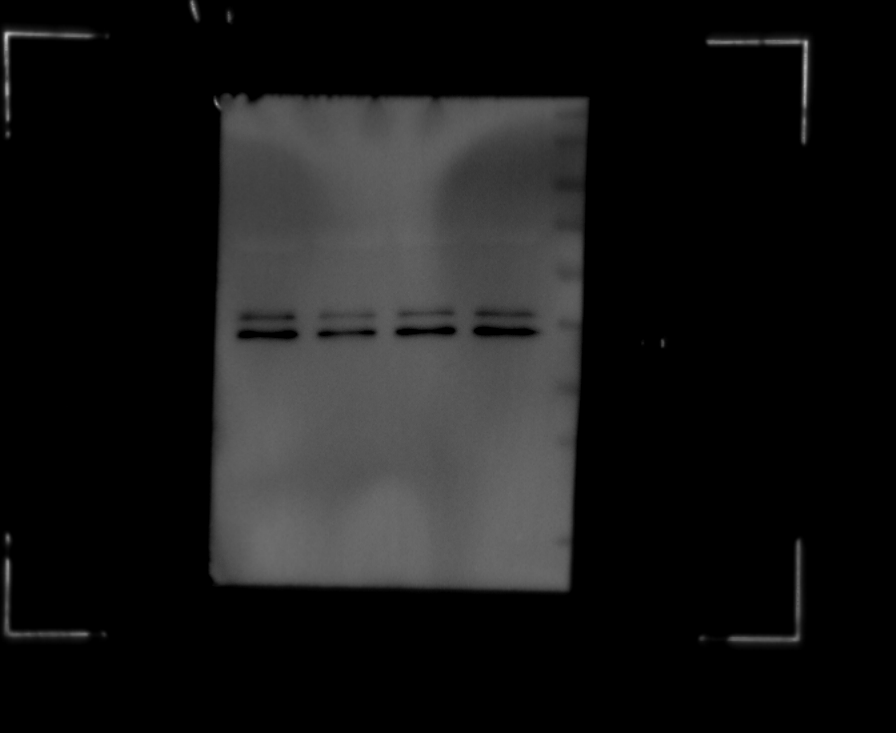

Supplement: Data S1. Original Western blot data [file mmc2.zip › WB原始图/iSicence Fig7B-Erk.tif]

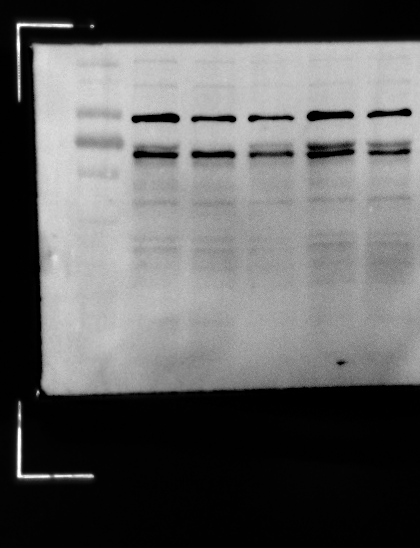

Supplement: Data S1. Original Western blot data [file mmc2.zip › WB原始图/iSicence Fig7B-TRAF6.tif]

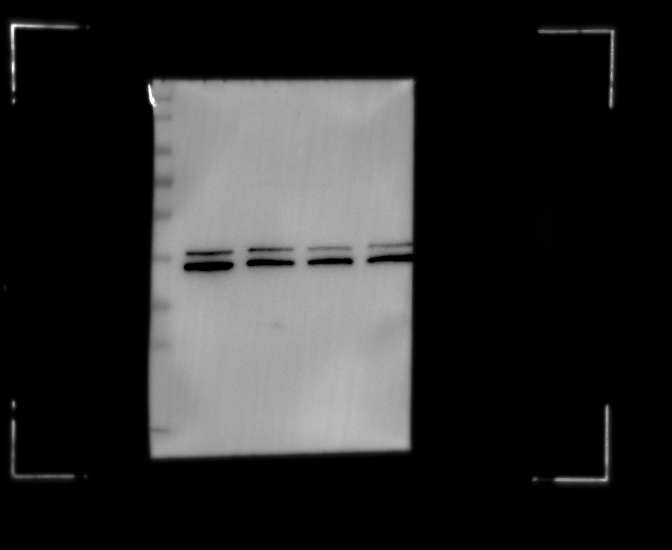

Supplement: Data S1. Original Western blot data [file mmc2.zip › WB原始图/iSicence Fig7F-Erk.tif]

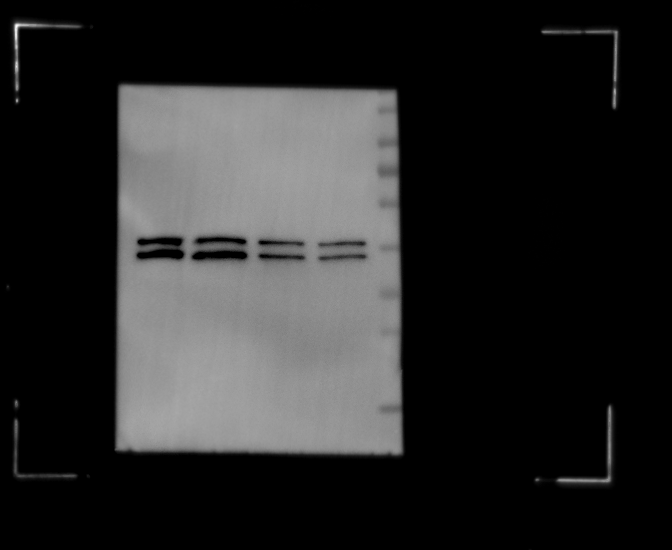

Supplement: Data S1. Original Western blot data [file mmc2.zip › WB原始图/iSicence Fig7F-p-Erk.tif]

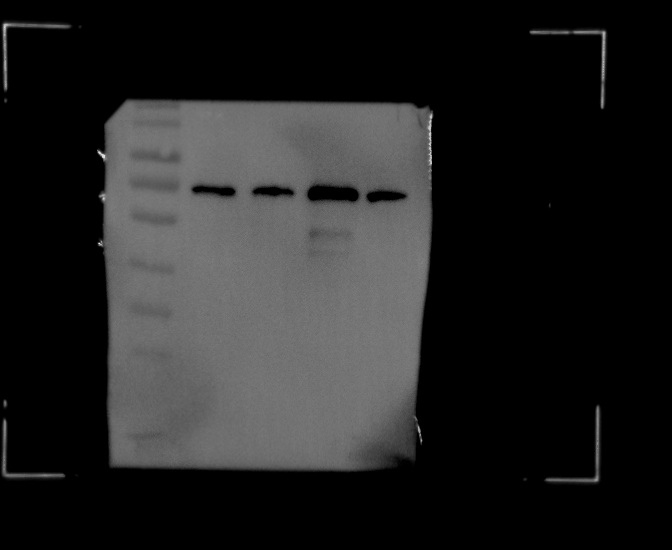

Supplement: Data S1. Original Western blot data [file mmc2.zip › WB原始图/iSicence Fig7F-p-p65.tif]

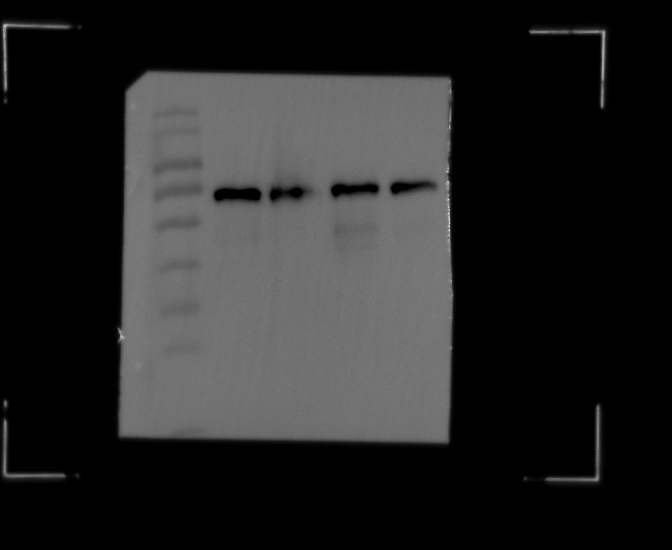

Supplement: Data S1. Original Western blot data [file mmc2.zip › WB原始图/iSicence Fig7F-p65.tif]

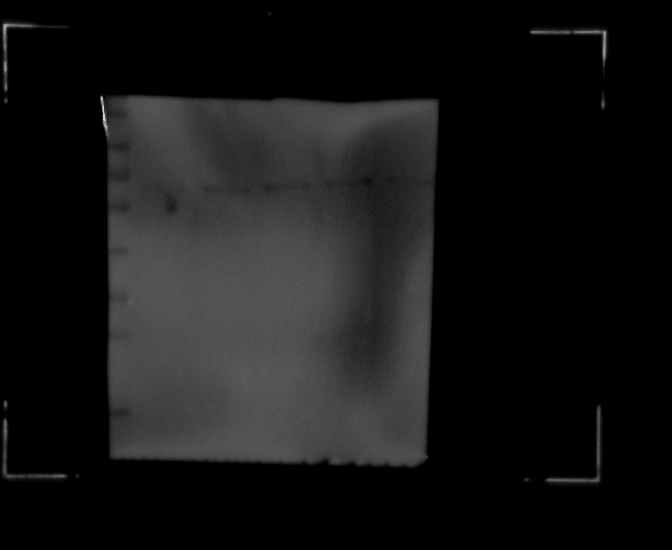

Supplement: Data S1. Original Western blot data [file mmc2.zip › WB原始图/iSicence Fig7F-TRAF6.tif]

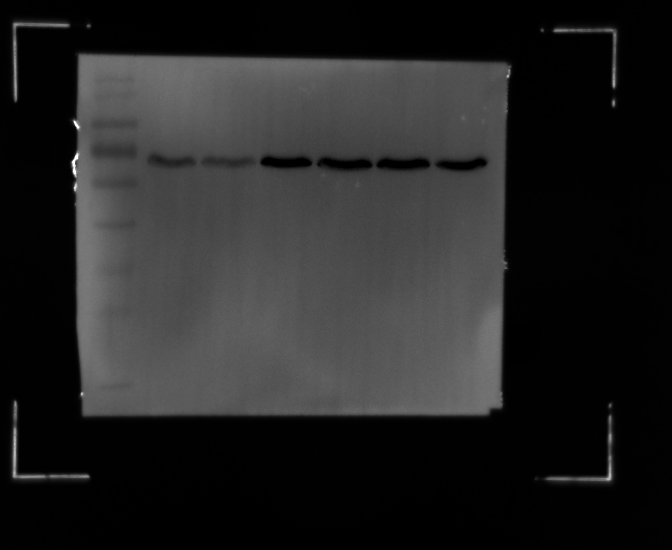

Supplement: Data S1. Original Western blot data [file mmc2.zip › WB原始图/iSicence SFig5A-p-p65.tif]

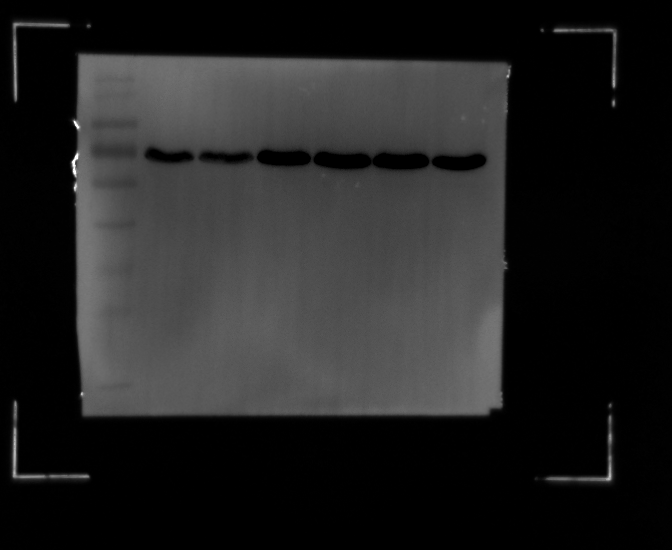

Supplement: Data S1. Original Western blot data [file mmc2.zip › WB原始图/iSicence SFig5A-p65.tif]

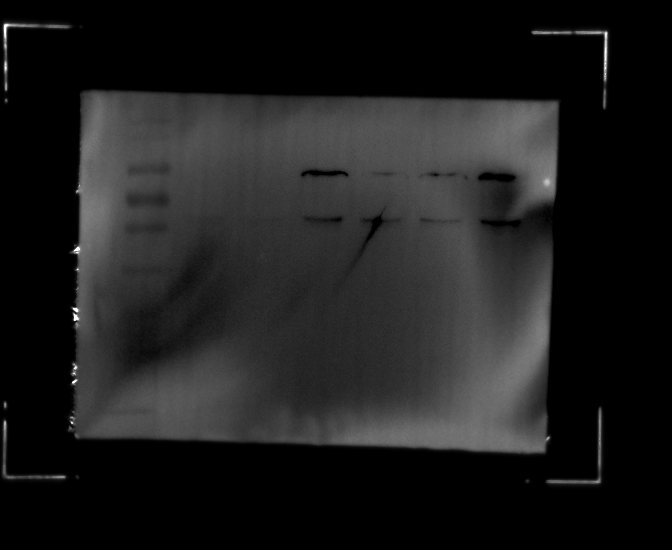

Supplement: Data S1. Original Western blot data [file mmc2.zip › WB原始图/iSicence SFig5A-TRAF6.tif]
